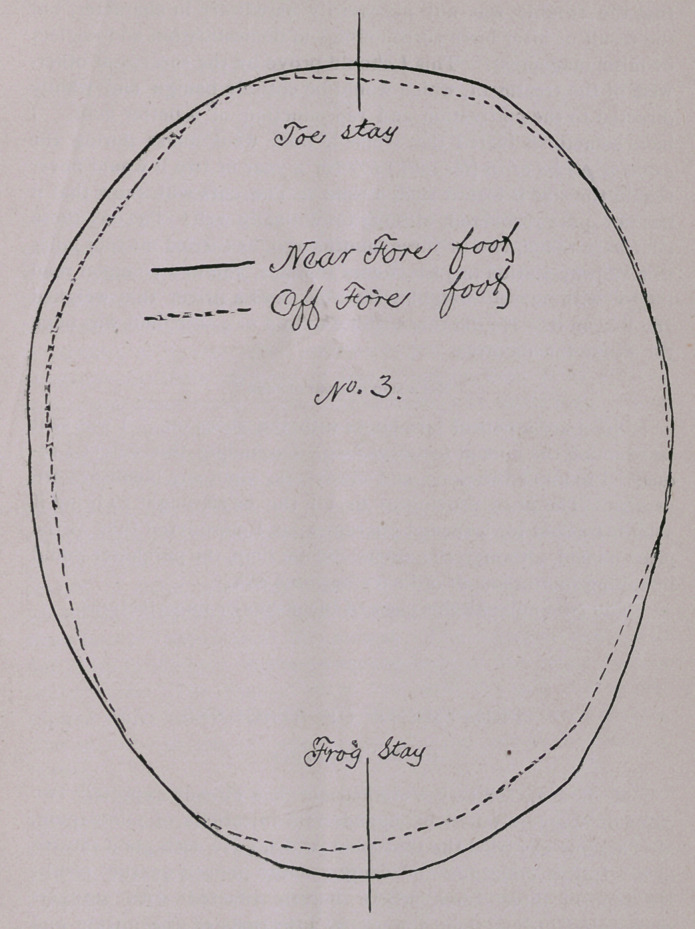# A Study in Horse Shoeing

**Published:** 1894-01

**Authors:** Williamson Bryden


### A STUDY IN HORSE SHOEING.

### SIZE, FORM OR SHAPE AND QUALITY OF THE HORSE'S HOOFS.

By Williamson Bryden, V.S.

In the study of these features of the horse's hoofs, we often meet with points of interest and having important bearings on the technical work of shoeing the horse's hoofs. Some one has said that "shoeing (the horse's hoofs) is a necessary evil." What nonsence! When properly performed it cannot be otherwise than a benefit, excepting from accident or when neglected in the stable.

I herewith submit to you two (2) pairs of hind hoofs *unshod* and one pair of fore hoofs *shod.*

No. 1 pair, *near* and *off* hind hoofs. This horse was owned by an aged couple; he was a rather moderate beast, about *13 years old*, or, as Dr. Holmes would possibly say, 13 years *young*; had always done his work well enough to suit till some six months before I was called, when the off hock became swollen to twice its previous size; he was very lame and a veterinary surgeon had been called in and had blistered his hock two or three times, but without improving the case. I was then called in, and on examination discovered that the *off* hoof was much smaller than the other, which was not a very good one either. A horseshoer being near by, I suggested that the horse be taken to the forge, where I reduced the hoofs and found them as the cuts indicate; by laying one on top of the other the difference in *size* and *shape* will be readily observed. Vide No. 1.

The horse was sent home very much better and able to do a little slow driving. I did not see the case again, but on meeting the horseshoer, an excellent mechanic, he informed me that after being three times shod he completely recovered; indeed, he had been able to perform his work from the time of my visit.

No. 2 pair, near and off hind hoofs of a young 4 year-old stallion. He was standard bred, a very desirable combination centered in him, and he had wonderful vigor and speed; much was expected of him until he was about two years old, when he became slightly lame on the off leg. The lameness was mostly a hitch. He was *let up* and merely *exercised* for two years. I was then called. On examination I discovered that the heel of the *off* hoof was narrower than the other; the shoe was worn most on the outside, toe and heel, the hoof being smaller than the *near* one. There was a slight puff above the ankle and an enlargement at the seat of bone spavin, due probably to the extra strain thrown on the perforans above the ankle and on the metatarsal flexor at its insertion at the hock. These strains come from several causes, whenever the hoof begins to colapse from improper environment after birth, adverse tissue changes within the hoof begin to take place; this disturbance reacts on the tendons, muscles, etc., of the leg above; some of these become shorter than others, and the leg is thrown out of harmony itself and with the other also; *the angles* formed by the various bones of the leg also causes certain muscles, tendons, etc., to be placed at greater disadvantage than others. He was then driven, when his *hitch* was very conspicuous; one hip was closer to the shaft than the other; the skin over the head of femur and its articulation *wrinkled* considerably when made to travel; the hip muscles were smaller than on the other side; the tail swerved just perceptibly to the derelict side and the crest of the ilium projected.

I then informed the owners that I considered the case one that could be much improved, perhaps entirely cured, but that it would take nine months or more to accomplish the repair. This being satisfactory I showed them what I intended to prove; that that the trouble was not necessarily hereditary in character, but the result of after birth surroundings in domestication and neglect or mismanagement. This I should prove by the success or otherwise of the treatment, which would be entirely benign and wholly directed to the cultivation and reproduction of a better hoof. I have sometimes feared that when a hoof went amiss during the *growing period of its* (*the colts'*) *life* for a year or two it would never regain the size it lost at such a time. The cuts will show that it has not quite recovered, although practically well. The day these cuts were taken, two or three months ago, he carried me four miles to a railway station in just twenty minutes, and in a common road buggy with myself weighing 225 lbs., and a driver that weighed 160 lbs., in it. He did this without a limp or a skip, and was none the worse for his drive.

"Not a step was out of tune."

No. 3 are a pair of fore hoofs with the shoes on. I was sent to examine the animal for *Soundness*. On finding this defect I requested to know the price, as I always do when any blemishes are present. The price proving to be all the beast ought to bring if sound, I asked for a special guarantee, as I considered that at the price named, although the animal did not limp, the purchaser would be taking more than an ordinary business risk.

The cuts indicate this class of blemishes or imperfections.

## Figures and Tables

**No. 1. f1:**
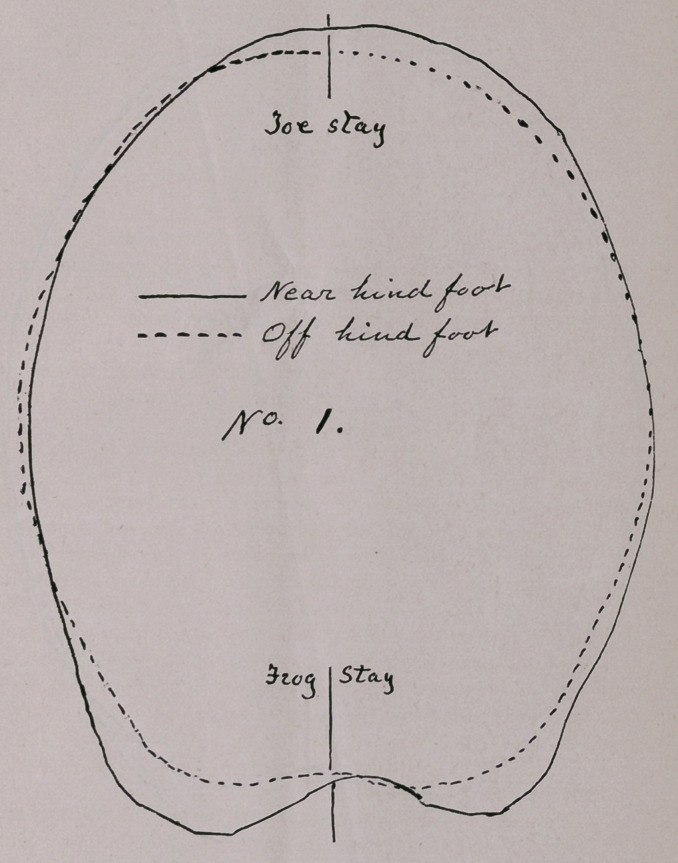


**No 2. f2:**
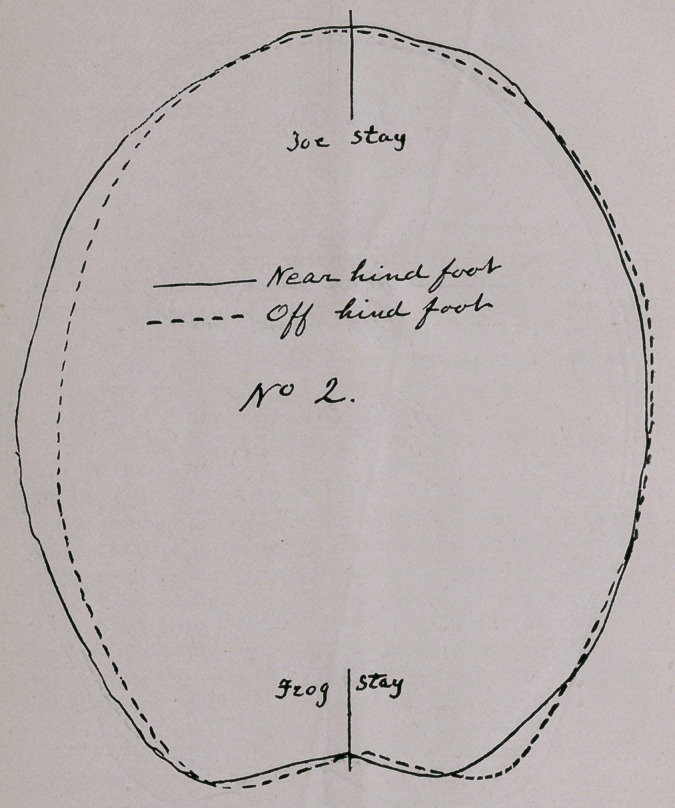


**No. 3. f3:**